# Analysis of a Two-Stage Magnetic Precession Gear Exploiting 3D Finite Element Method

**DOI:** 10.3390/ma18235277

**Published:** 2025-11-22

**Authors:** Lukasz Macyszyn, Cezary Jedryczka, Michal Mysinski

**Affiliations:** 1 Faculty of Mechanical Engineering, Poznan University of Technology, pl. Marii Sklodowskiej-Curie 5, 60-965 Poznan, Poland; 2 Faculty of Control, Robotics and Electrical Engineering, Poznan University of Technology, pl. Marii Sklodowskiej-Curie 5, 60-965 Poznan, Poland; cezary.jedryczka@put.poznan.pl (C.J.); michal.mysinski@doctorate.put.poznan.pl (M.M.)

**Keywords:** magnetic precession gear, 3D finite element method (FEM), permanent magnets, torque, magnetic flux distribution

## Abstract

The paper presents the results of numerical simulations carried out to investigate the influence of selected geometric parameter–precession angle and dimensions of the magnetic circuit of a two-stage magnetic precession gear on the magnetic torques acting on its active components. The operating principle of the proposed gear and the developed numerical model based on the 3D finite element method (FEM) are discussed. The study focuses on the effects of air gap length, magnet dimensions, pole pitch coverage and precession angle. The results confirm a strong correlation between these parameters and the transmitted torque, providing valuable guidelines for the optimal design of high-torque, compact and efficient magnetic precession gears.

## 1. Introduction

Magnetic gears (MGs) offer several advantages over traditional mechanical gears, including reduced maintenance, high reliability, and inherent overload protection due to their contactless operation. These benefits make MGs particularly suitable for applications requiring low maintenance, such as marine propulsion and wind power generation [1,2,3]. One of the primary types of MGs is the coaxial magnetic gear (CMG), which is known for its high torque transmission capability [4,5]. Another innovative approach involves integrating magnetic gears into the structure of permanent magnet synchronous machines, creating a more compact and efficient drive unit. This integration can lead to additional mass savings and improved performance in applications requiring high torque density [1]. Magnetic gears also offer advantages in terms of noise and vibration reduction as well as reliability improvement. Unlike mechanical gears, MGs do not require lubrication and are less prone to wear and tear, making them ideal for applications where maintenance is challenging or costly [6,7].

However, magnetic gears face issues such as severe eddy current and hysteresis losses in the magnetic circuit and centrifugal forces at high speeds, which can lead to efficiency drops and potential damage [4]. Magnetic gears also have a much lower torsional stiffness than mechanical gears. This low stiffness results in long-lasting oscillations during transient changes in speed and load [8,9,10,11]. The torsional stiffness of magnetic gears is significantly lower than the meshing stiffness of mechanical gears, which affects their dynamic performance [12,13].

**Figure 1:**
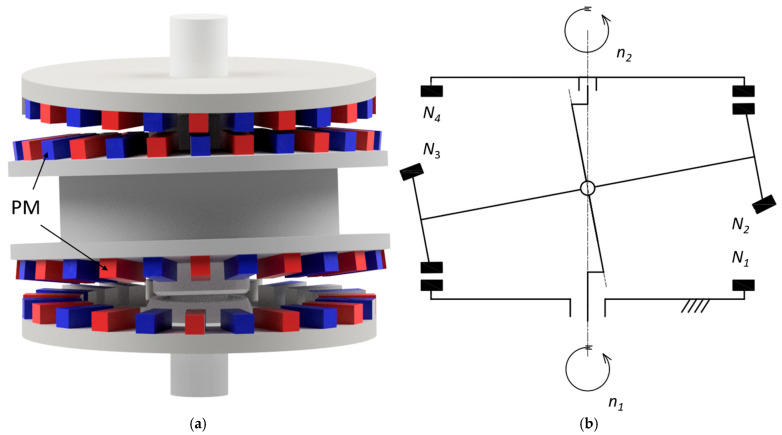
Two-stage magnetic precession gear CAD model (**a**) and kinematic scheme (**b**): PM—permanent magnet, *n*_1_—input speed, *n*_2_—output speed, *N*_k_ number of magnets on ring k (k = 1, 2, 3, 4).

The article is focused on analysis of magnetic precession gears (MPG), which offer several advantages over traditional mechanical gearboxes and other types of MGs, primarily due to their unique design and operational principles that leverage magnetic forces for torque transmission. One of the key benefits is their ability to achieve greater transmission ratios compared to other magnetic gears, thanks to their specific geometrical and kinematical design, which allows for optimal gear ratio configurations [14]. The transmission ratio for this type of gear may be obtained in the range of 1/80 ÷ 1/10,000 [15]. Thanks to these advantages, magnetic precession gear could be applied, for example, in tidal and wave energy converters, low-speed direct-drive wind turbines, hermetic pumps for corrosive or radioactive fluids, bioreactors and pharmaceutical mixers, and precision positioning systems in space and underwater robotics. Figure 1 presents the CAD model and the kinematic diagram of the MPG.

## 2. Materials and Methods

The research conducted aims to understand the effect of geometric parameters and the dimensions of the magnetic circuit on the magnetic torques acting between the magnetically active components of the MPG. The transmitted torque constitutes a fundamental performance parameter of an MG. Therefore, the design process of the newly proposed gearbox required the development of a reliable model capable of accurately determining torque values for specific geometric and magnetic configurations. In magnetic gears, torque transmission is governed by the distribution of the magnetic field within the air gaps separating the magnetically active components of the MPG. To investigate this phenomenon, a comprehensive analysis of the magnetic precession gear performance was conducted using a finite element model of the magnetic field, implemented in the Ansys Maxwell 3D professional FEM environment.

**Figure 2:**
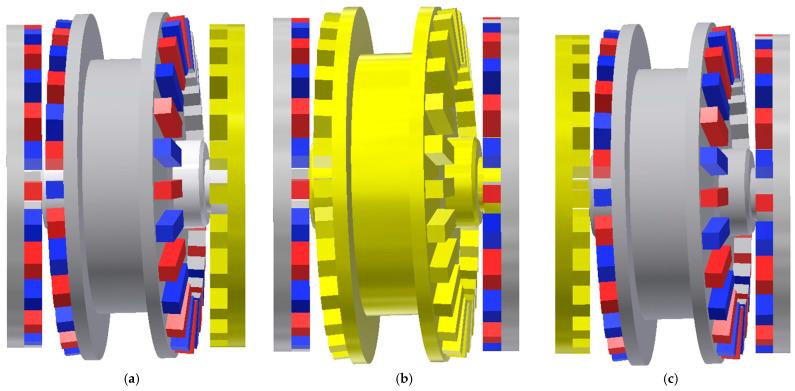
Geometries for calculating torques: (**a**) output ring, (**b**) intermediate ring (**c**) immovable ring.

The examined system incorporates two air gaps that directly participate in torque transmission. Owing to the presence of non-coaxial rotational axes, torque evaluation was conducted for three different geometrical arrangements, as presented in Figure 2.

**Figure 3:**
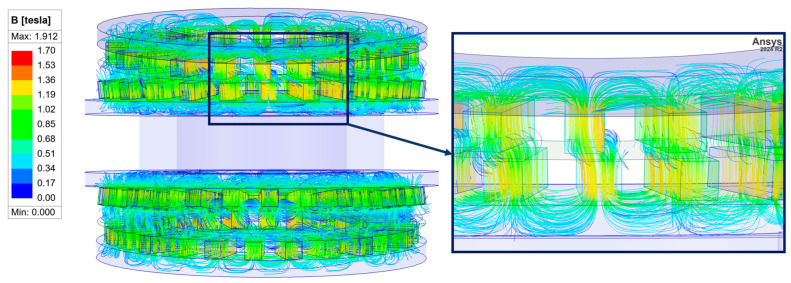
Example of magnetic flux distribution in the air gap between an output ring and an intermediate ring.

For the determination of torque acting between the interacting elements of the designed gear, the Maxwell stress tensor method was employed [16]. The finite element mesh comprised approximately 897,000 tetrahedral elements, with nonlinear magnetic properties of the core material duly accounted for. At the present stage of the study, the effects of eddy currents have been disregarded. An example of the magnetic flux distribution within the air gap between the immovable outer ring and the intermediate ring is illustrated in Figure 3. Detailed description of the developed numerical model of the MPG can be found in [17]. Accuracy of the model was verified experimentally on the prototype of the gear ratio 144 and precession angle 6°. For all analyzed cases, root mean square error does not exceed 10% of the maximum transmitted torque which is considered a good agreement between model and experiment.

**Figure 4:**
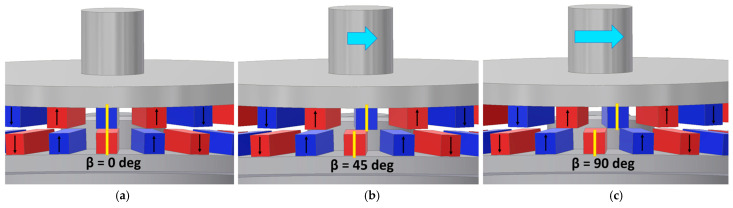
Illustration of the load angle changes upon the load torque: (**a**) β = 0 deg; (**b**) β = 45 deg; (**c**) β = 90 deg.

As highlighted in the introduction, a characteristic property of magnetic gears is their significantly lower stiffness relative to conventional mechanical gears. Consequently, variations in load torque lead to changes in the mechanical angle between the movable components, as illustrated in Figure 4. This behavior is analogous to the correlation between load torque and the internal load angle observed in synchronous motors [18,19]. The blue arrows in Figure 4 represent both the magnitude and direction of the applied load torque.

Two load angles can be defined for the studied MPG. The load angle between the immovable ring and the intermediate ring will be marked as β_1_ while the load angle between the intermediate ring and the output ring will be depicted as β_2_.

The next chapter presents the results of FEM simulations aimed at examining the influence of selected geometric parameter–precession angle and dimensions of the magnetic circuit on the magnetic torques acting on the magnetically active components of the MPG, namely the output ring (*T*_out_), the immovable ring (*T*_base_), and the input shaft together with the intermediate ring (*T*_in_). The developed numerical model of the magnetic field in the studied gear enables an independent evaluation of the effect of each parameter. Section 3.1 addresses the impact of air gap lengths. The investigations reported in Section 3.2 and Section 3.3 focus on the influence of the dimensions and shape of the magnets. Finally, the results presented in Section 3.4 allow for an assessment of the effect of the precession angle.

## 3. Results and Discussion

### 3.1. Examination of the Influence of Air Gap Lengths

**Figure 5:**
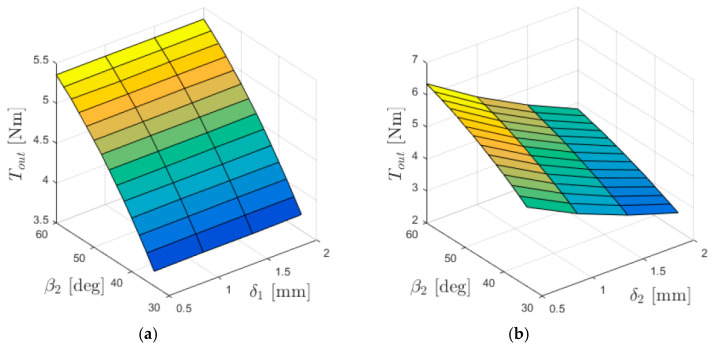
Output torque *T*_out_ as a function of the internal load angle β_2_ and: (**a**) δ_1_ (for δ_2_ = 1 mm); (**b**) δ_2_ (for δ_1_ = 1 mm).

**Figure 6:**
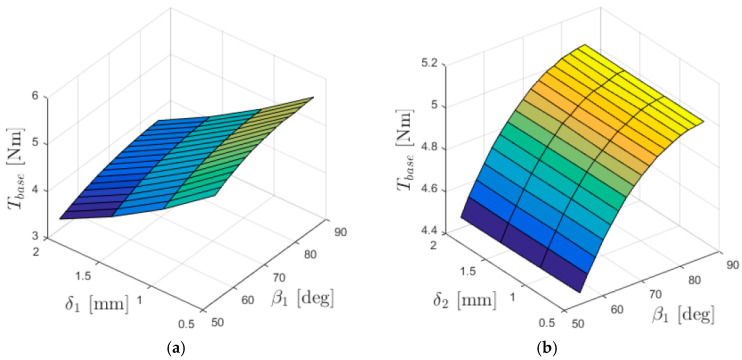
Torque acting on the immovable ring *T*_base_ as a function of the internal load angle β_1_ and: (**a**) δ_1_ (for δ_2_ = 1 mm); (**b**) δ_2_ (for δ_1_ = 1 mm).

**Figure 7:**
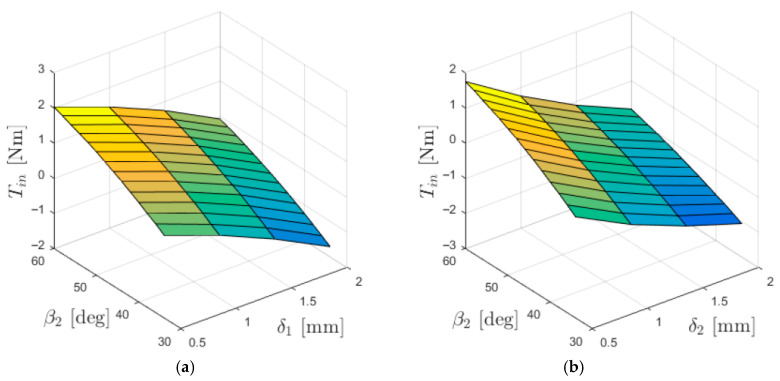
Torque acting on the input ring *T*_in_ as a function of the internal load angle β_1_ and: (**a**) δ_1_ (for δ_2_ = 1 mm); (**b**) δ_2_ (for δ_1_ = 1 mm).

The relationships between the torques acting on individual gear rings and the lengths of the air gaps (between the immovable ring and the intermediate ring—δ_1_, and between the intermediate ring and the output ring—δ_2_) are presented in Figure 5, Figure 6 and Figure 7.

Assuming proper design of the magnetic circuit, namely ensuring sufficient cross-sections of the ferromagnetic yokes formed by ferromagnetic discs of the MPG, the length of the air gap has a decisive influence on the reluctance of the magnetic circuit of the considered gear. In analytical methods, for simple magnetic circuits, the magnetic reluctance of the air gap is calculated according to the following relation:

(1) $$R_{\mu \delta }=\frac{l}{\mu_{0}S}\;$$

where *l* is the length of the air gap (the path of the magnetic flux in the circuit), μ_0_—the magnetic permeability of vacuum, *S*—the cross-sectional area through which the flux passes.

In the considered transmission, defining the length *l* and the area *S* is complicated due to the complex nature of the magnetic circuit structure. Nevertheless, obviously an increase in the air gap length will lead to an increase in the reluctance for the magnetic flux. In turn, the increase in reluctance results in a decrease in the magnetic flux density in the air gap, which translates into a reduction in the interaction force between the magnets and, consequently, a decrease in torque.

From the graphs shown in Figure 5, it can be concluded that the output torque *T*_out_ depends on the length of the air gap δ_2_, while it is independent of δ_1_. Reducing the length of the air gap between the intermediate and the output ring results in an increase in the torque acting on the output ring at a given internal load angle.

From the graphs shown in Figure 6, it can be inferred that the torque acting on the immovable ring *T*_base_, depends on the length of the air gap δ_1_, whereas it is independent of δ_2_. Similarly to the relationship of *T*_out_ with δ_2_, the value of *T*_base_ increases as the air gap length decreases for a given internal load angle.

The dependence of the input torque *T*_in_ on the lengths of the air gaps between the cooperating rings (Figure 7) is significantly more complex than in the case of *T*_base_ and *T*_out_. The input torque is influenced by the distribution of the magnetic field in both air gaps, and therefore its value is affected by the lengths of both δ_1_ and δ_2_.

### 3.2. Investigation of the Influence of Magnet Dimensions

**Figure 8:**
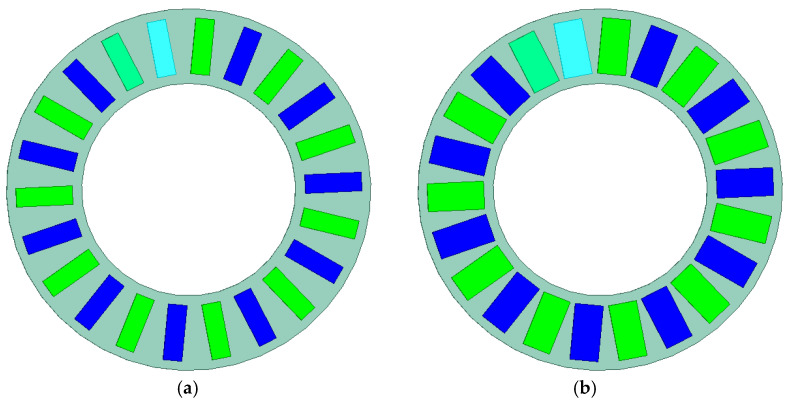
Immovable ring with magnets of 5 mm (**a**) and 7.5 mm (**b**) width.

**Figure 9:**
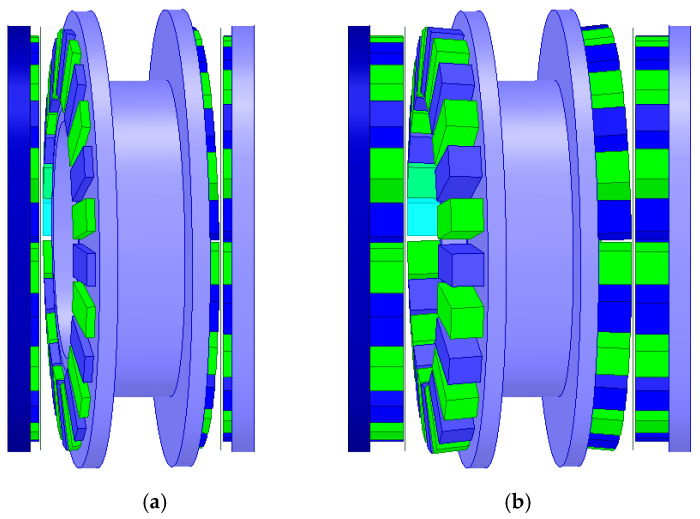
Geometry of MPG with magnets of 7.5 mm width and heights of 2 mm (**a**) and 8 mm (**b**).

A study was also conducted on the influence of the width and height of the magnets on the torque acting on the output ring of the gear. First, with a constant magnet height of 5 mm, calculations were performed for magnet widths ranging from 5 to 7.5 mm. Then, for the maximum magnet width (7.5 mm), the effect of magnet height (ranging from 2 to 10 mm) on the generated output torque was investigated. Figure 8 shows the geometry of the immovable ring with magnets of 5 mm width (Figure 8a) and 7.5 mm width (Figure 8b), while Figure 9 presents the geometry of the MPG with magnets of 7.5 mm width and heights of 2 mm (Figure 9a) and 8 mm (Figure 9b).

**Figure 10:**
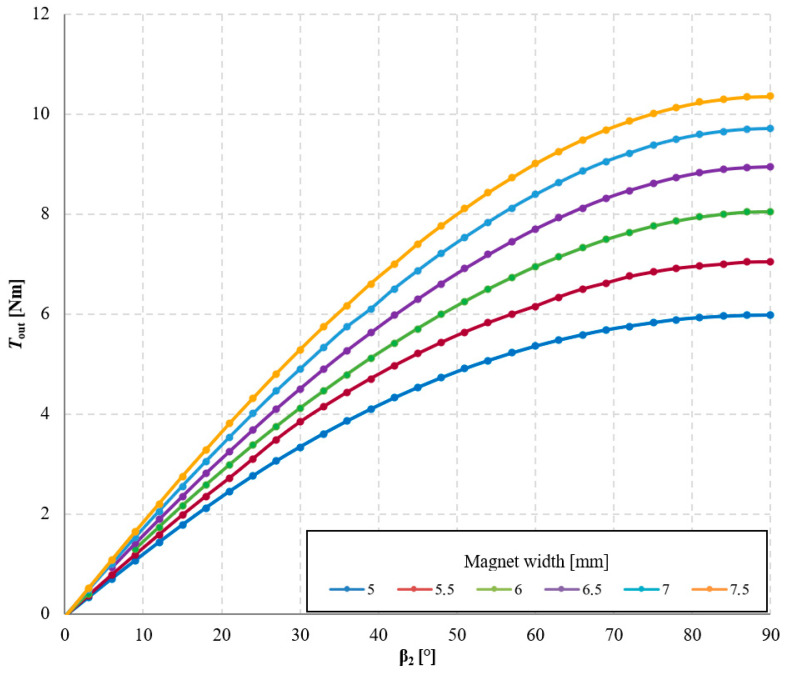
Torque acting on the output ring, *T*_out_, as a function of the internal load angle β_2_ and magnet width, for symmetric air gaps δ_1_ = δ_2_ = 1 mm, with a constant angle β_1_ = 30° and a fixed magnet height of 5 mm.

**Figure 11:**
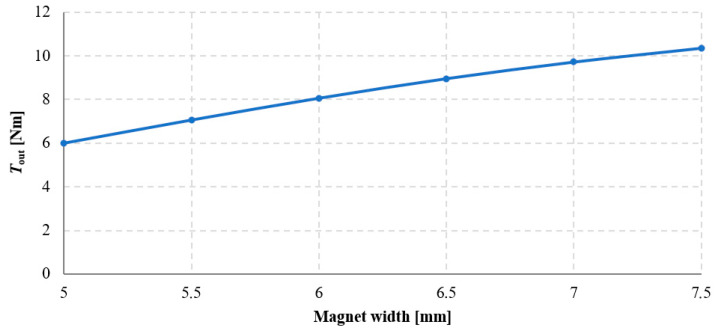
Torque acting on the output ring, *T*_out_, as a function of magnet width for an angle β_2_ = 90° with a constant magnet height of 5 mm.

The results of the study on the influence of magnet width on the torque acting on the output ring of the transmission are presented in Figure 10 and Figure 11.

**Figure 12:**
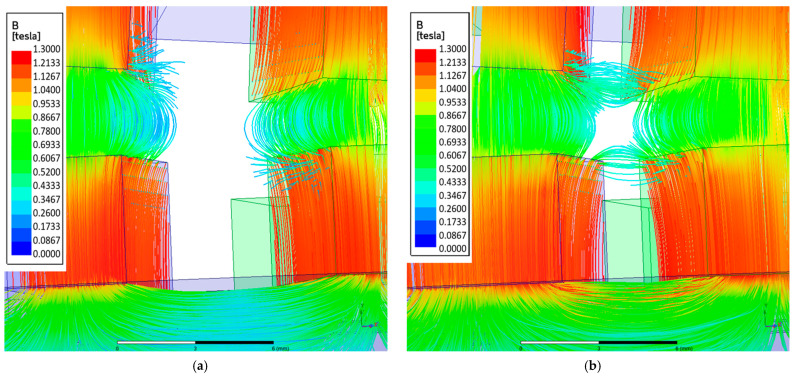
Magnetic flux distribution in the air gap between the immovable ring and the intermediate ring for magnet widths: (**a**) 5 mm; (**b**) 7.5 mm.

Based on these results, it can be concluded that as the magnet width increases, the torque acting on the output ring also increases. The torque acting on magnetically active components of the MPG is calculated as an integral of the Maxwell stress tensor components over the integration surface located in the air region surrounding the magnetically active materials of each element. Therefore, the larger the magnetically active surface, the greater the achievable torque. However, this relationship is nonlinear, and the cause of this nonlinearity is the presence of flux leakage. This phenomenon occurs when part of the magnetic flux flows to an adjacent magnet on the same ring, thereby reducing the main flux directed toward the air gap. The occurrence of flux leakage can be observed in the magnetic flux distributions shown in Figure 12. It is evident that in the case of wider magnets, the flux leakage is significantly greater. Consequently, increasing the magnet width by 0.5 mm—from 5 mm to 5.5 mm—results in an increase of *T*_out_ by approximately 1.07 Nm, whereas an identical increase in width from 7 mm to 7.5 mm results in a smaller torque increase torque *T*_out_ about 0.64 Nm.

Referring to the classical magnetic circuits of electrical machines with permanent magnets, the concept of pole pitch coverage by the permanent magnet (magnet span relative to the pole pitch) [20] can be applied here. It should be noted that when magnets of 5 mm width are used, the pole pitch coverage on individual rings is approximately 50%, whereas for 7.5 mm magnets it reaches about 75%. The reduction in the increment of useful torque for increased magnet width in the analyzed transmission configuration is related to the adopted shape of the permanent magnets. Higher values of pole pitch coverage can be achieved after modifying the magnet shape, described in Section 3.3.

**Figure 13:**
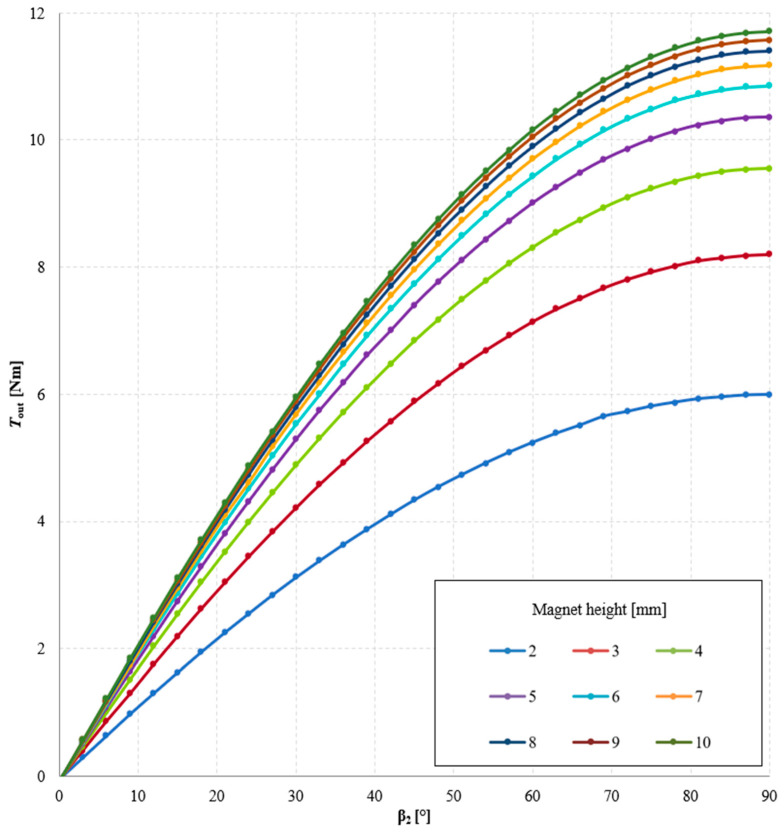
Torque acting on the output ring *T*_out_ as a function of the internal load angle β_2_ and magnet height, for symmetric air gaps δ_1_ = δ_2_ = 1 mm, with a constant angle β_1_ = 30° and a fixed magnet width of 7.5 mm.

**Figure 14:**
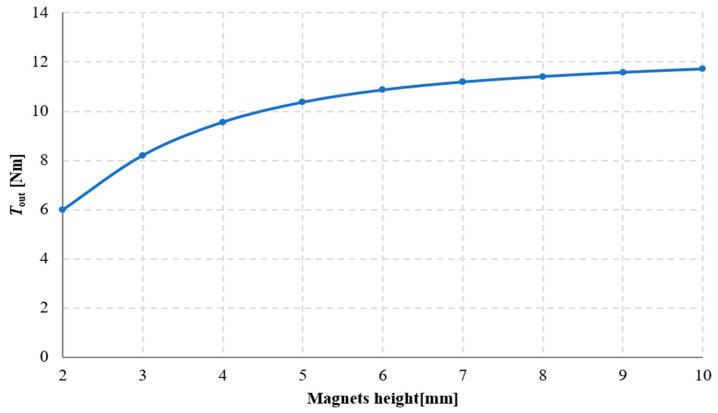
Torque acting on the output ring *T*_out_ as a function of magnet height for an angle β_2_ = 90° with a constant magnet width of 7.5 mm.

The results of the study on the influence of magnet height on the torque acting on the output ring of the gear are presented in Figure 13 and Figure 14. From these results, it can be concluded that the torque acting on the output ring increases with magnet height. This is directly related to the increase in the magnetomotive force of the magnet. In magnetic circuits with permanent magnets, the magnet height—defined as the dimension of the magnet along the magnetization direction—has a direct impact on the value of the magnetomotive force. In general, the magnetomotive force of a magnet can be expressed as:

(2) $$\Theta =H_{m}\cdot \;l$$

where Θ is the magnetomotive force, *H*_m_—the magnetic field strength for the considered material and operating point, and *l*—the magnet height in the direction of magnetization.

**Figure 15:**
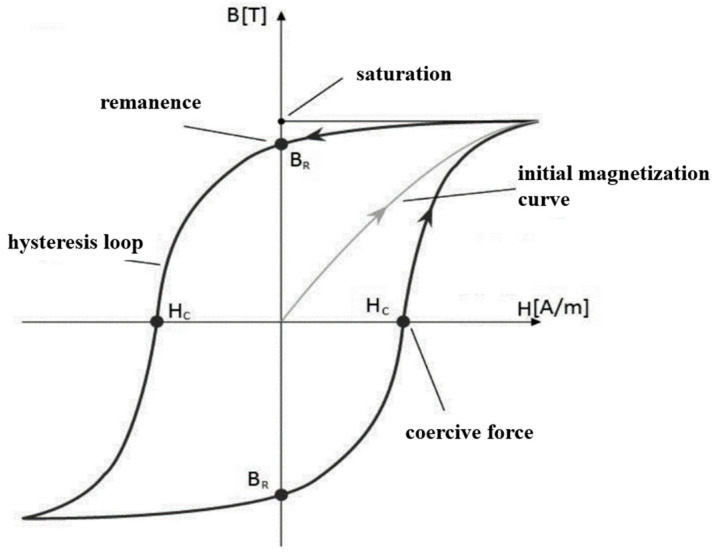
Example of hysteresis loop of ferromagnetic material [21].

Figure 15 presents an exemplary *B–H* hysteresis loop, which describes the behavior of a ferromagnetic material in a magnetic field. When a ferromagnet is placed in an external magnetic field of increasing intensity *H*, the magnetic flux density *B* rises until saturation is reached. When the external field intensity *H* is then reduced to zero, the magnetic flux density *B* attains the value *B*_r_ (referred to as remanent flux density or remanence). Subsequently, when the field direction is reversed, the flux density decreases until it reaches zero at a field intensity equal to *H*_c_. Further increasing the field intensity again leads to a rise in flux density (in the opposite direction) until saturation is reached [21].

Thus, a permanent magnet in a closed ferromagnetic circuit (without an air gap and assuming infinite permeability of the ferromagnetic core) has a remanent flux density *B*_r_, and its operating point is indicated in Figure 15 by point *a*. When an air gap is introduced into the circuit, the magnetic reluctance of the circuit increases, the magnetic flux density in the core decreases, and the operating point shifts along the hysteresis loop to point *b* [22].

According to Ampère’s law, the magnetomotive force Θ in a given magnetic circuit balances the magnetic voltages resulting from the magnetic reluctance and the generated magnetic flux ϕ. Neglecting the reluctance of the core (assuming that the magnetic permeability of the ferromagnetic core tends to infinity), it can therefore be assumed that the value of the magnetic flux in the considered circuit depends on the magnetomotive force produced by the permanent magnet and on the magnetic reluctance of the air gap *R*_μδ_. This relationship can thus be expressed by the following equation:

(3) $$H_{m}\cdot l=\;-\;R_{\mu \delta }\cdot \phi .$$

The magnetic flux, in turn, depends on the active surface area of the magnet *S_m_* and the magnetic flux density *B_m_* at the operating point of the magnet:

(4) $$\phi =B_{m}\cdot S_{m}$$

With a constant air gap length (and thus a constant *R*_μδ_, increasing the magnet height reduces the magnetic field strength in the permanent magnet *H*_m_, and consequently, the magnetic flux density in the analyzed circuit increases in accordance with the recoil line [23]. This naturally leads to an increase in the torque acting on the output ring. However, the dependence of torque on magnet height *l* is nonlinear, since as *l* increases, the operating point approaches the value of *B*_r_, and results in worsening exploitation of PM material (in terms of maximal energy product *BH*_max_ [24]. The analysis presented in Figure 15 shows that the difference in the maximum value of *T*_out_ when the magnet height increases from 2 mm to 3 mm is approximately 2.2 Nm, whereas increasing the magnet height from 9 mm to 10 mm results in a rise in the maximum *T*_out_ of only about 0.1 Nm.

### 3.3. Investigation of the Influence of Magnets Shape

**Figure 16:**
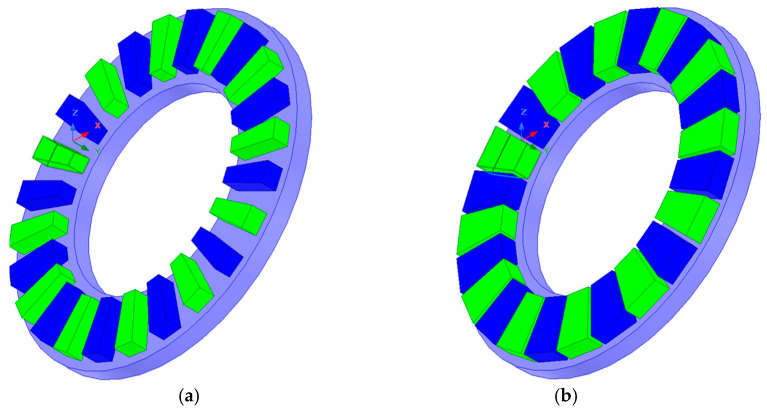
Immovable ring with magnets of a modified shape, providing a pole pitch coverage factor by the magnet equal to: (**a**) 0.5; (**b**) 0.9.

In the simulation studies conducted so far, rectangular permanent magnets with standard dimensions, identical to those used in the prototype, were applied. When such magnets are arranged along the circumference of the ring, the distances between the edges of adjacent magnets are not constant—the magnets are closer to each other on the side facing the center of the ring. Changing the shape of the magnets to those shown in Figure 16 makes it possible to increase the pole pitch coverage with the permanent magnet. This ensures constant distances between the sides of adjacent magnets, which in turn reduces the value of flux leakage while maintaining the active surface area of the magnet.

**Figure 17:**
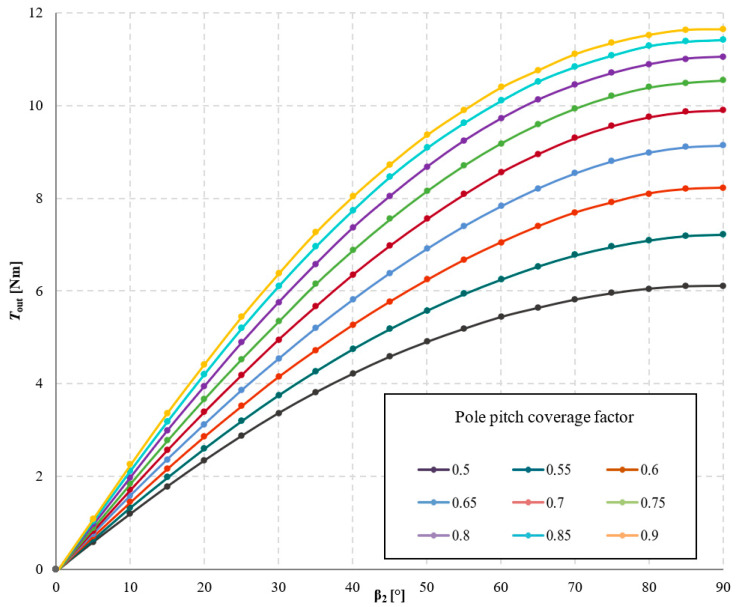
Torque acting on the output ring, *T*_out_, as a function of the internal load angle β_2_ and the pole pitch coverage factor of the permanent magnet, for symmetric air gaps δ_1_ = δ_2_ = 1 mm, with a constant angle β_1_ = 30° and a fixed magnet height of 5 mm.

**Figure 18:**
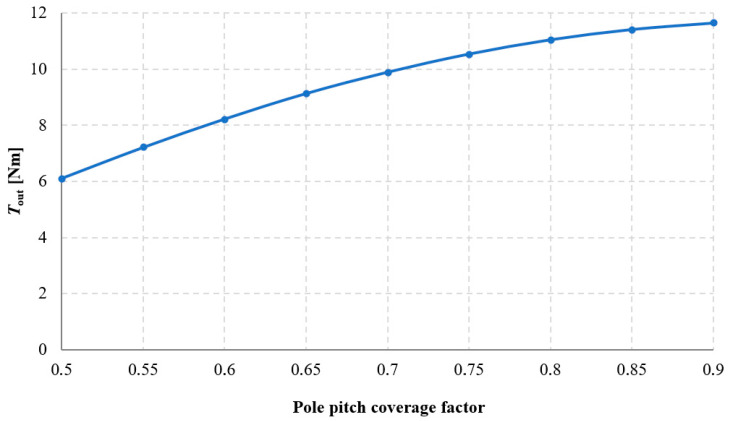
Torque acting on the output ring, *T*_out_, as a function of the pole pitch coverage factor of the permanent magnet for an angle β_2_ = 90°, with a constant magnet height of 5 mm.

**Figure 19:**
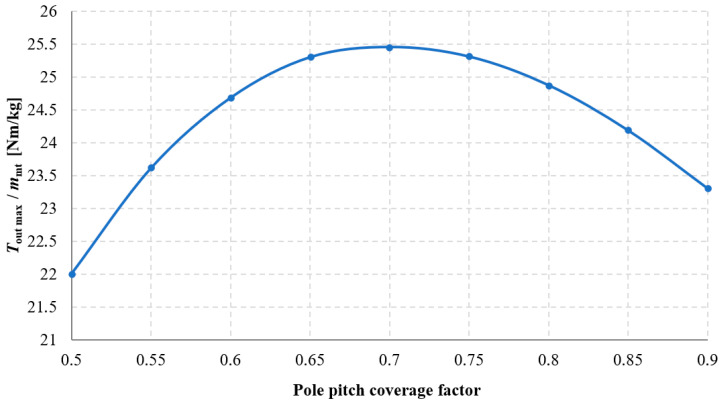
Ratio of *T*_out_ to the mass of the applied permanent magnets *m*_pm_ as a function of the pole pitch coverage factor of the permanent magnet, for β_2_ = 90° and a constant magnet height of 5 mm.

The influence of pole pitch coverage by the permanent magnet on the torque acting on the output ring *T*_out_ was investigated, and the results of these studies are presented in Figure 17 and Figure 18. Analyzing the obtained results, it can be stated that as the pole pitch coverage by the magnet increases, the value of *T*_out_ also increases. Consequently, the torque density transmitted by the gear also increases. However, this relationship is nonlinear, similarly to the case of changing the width of rectangular magnets, described in Section 3.2. It should be noted that permanent magnets constitute a very significant component of the overall cost of the transmission. Therefore, it was examined for which value of the pole pitch coverage factor the efficiency of permanent magnet utilization—understood as the ratio of the maximum obtainable torque to the mass of the applied permanent magnets—is most favorable. This relationship is shown in Figure 19. Hence, it should be considered whether, in some applications, it may be more advantageous to increase the distance between the magnets at the expense of achieving a lower transmitted torque density.

### 3.4. Investigation of the Influence of Precession Angle

**Figure 20:**
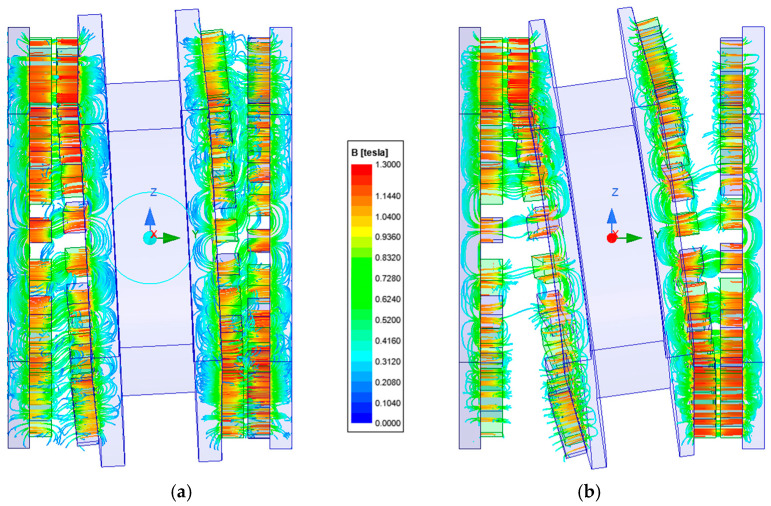
Magnetic flux distribution for a precession angle of: (**a**) 3°; (**b**) 6°.

**Figure 21:**
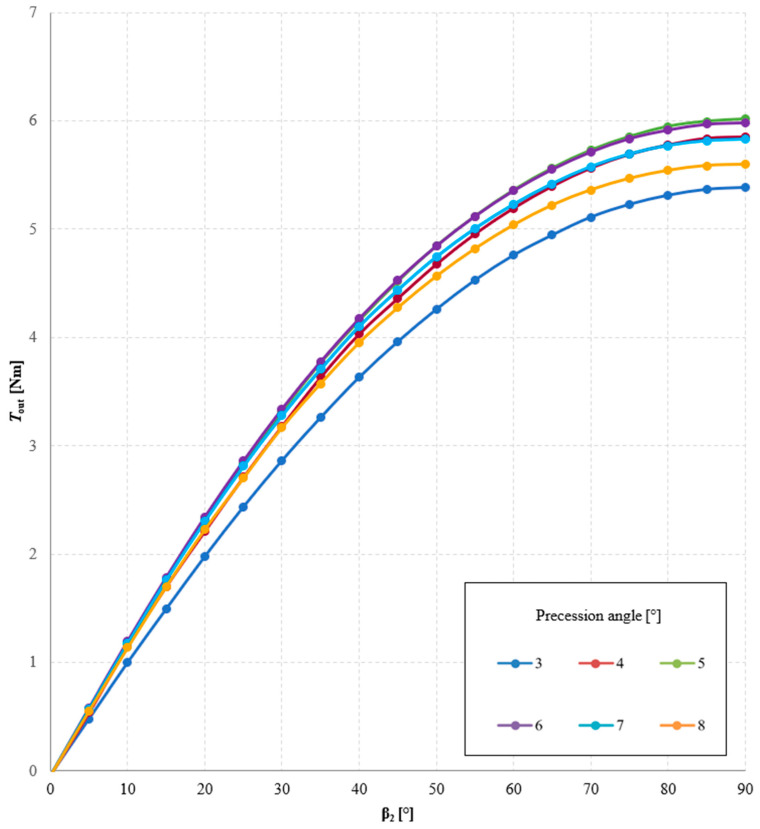
Torque acting on the output ring, *T*_out_, as a function of the internal load angle β_2_ and the precession angle, for symmetric air gaps δ_1_ = δ_2_ = 1 mm and a constant angle β_1_ = 30°.

**Figure 22:**
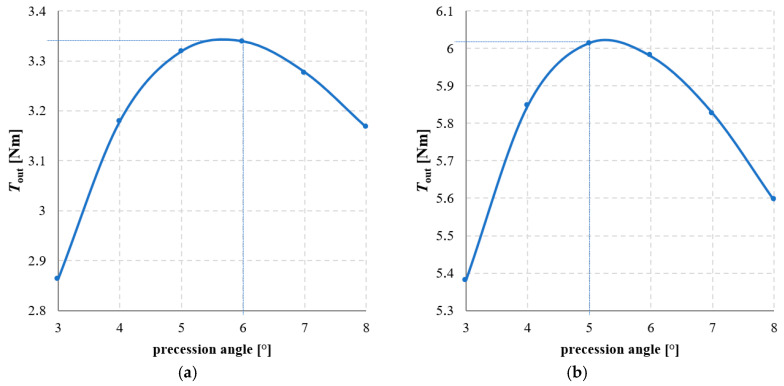
Torque acting on the output ring, *T*_out_, as a function of the precession angle for the internal load angle β_2_: (**a**) 30°; (**b**) 90°.

The precession angle, defined as the angle between the axis of the intermediate ring and the main axis of the gear, was also found to be a significant factor affecting the torque transmitted by the gear. Figure 20 presents the magnetic flux lines distribution for two different precession angles. Figure 21 and Figure 22 show the dependence of the torque acting on the output ring *T*_out_ on the precession angle. A too small precession angle reduces the air gap length between magnets that do not participate in torque transmission, causing some of them to generate torque in the opposite direction to that intended. Conversely, a too large precession angle decreases the magnetically active surface area between the cooperating rings, which also negatively affects the torque value.

In the analyzed MPG design with a gear ratio of 1/144 and magnets of dimensions 5 × 5 × 15 mm, the maximum values of *T*_out_ were obtained for precession angles of 5° and 6°, depending on the internal load angle β_2_. Analyzing the graphs presented in Figure 22, it can also be observed that decreasing the precession angle from its optimal value has a greater impact on the reduction of *T*_out_ than increasing the precession angle.

## 4. Conclusions

The conducted research enabled a comprehensive understanding of how the geometrical parameters and the magnetic circuit configuration of a two-stage magnetic precession gear affect the magnetic torques acting on its active components. The finite element analyses confirmed that variations in air gap length, magnet dimensions, pole pitch coverage, and precession angle significantly influence the torque transmission capability of the gear.

The results indicated that reducing the air gap length leads to higher transmitted torque. Increasing the magnet width and height also enhances the torque, though this dependence is nonlinear due to flux leakage and magnetic saturation. The analysis of impact of pole pitch coverage by the magnet was shown to be a crucial factor: higher coverage increases torque density but may reduce the effectiveness of magnet utilization, which is vital from both design and economic perspectives. The efficiency of permanent magnet utilization turned out to be the highest for the pole pitch coverage factor of 0.7. The precession angle was also identified as an important parameter. In the presented gearbox maximum torque was achieved for a precession angle between 5 and 6 degrees. Deviations from this value negatively impacted performance. In all of the analyses, the maximum torque was obtained for the output load angle close to 90 degrees.

Overall, the study highlights the importance of precisely selecting and adjusting geometrical parameters in magnetic precession gear design. By carefully balancing air gap dimensions, magnet geometry, pole pitch coverage and precession angle, it is possible to significantly improve torque transmission while ensuring efficient use of permanent magnets. These insights provide a valuable foundation for further development of high-torque-density, low-maintenance magnetic transmission systems suitable for demanding applications such as renewable energy and marine propulsion.

## Data Availability

The original contributions presented in this study are included in the article. Further inquiries can be directed to the corresponding author.

## Abbreviations

The following abbreviations are used in this manuscript:

FEM	Finite Element Method
MG	Magnetic gear
MPG	Magnetic precession gear
